# Editorial: Maternal and infant nutrition: impact on breast milk, infant gut microbiota and health development

**DOI:** 10.3389/fnut.2025.1707323

**Published:** 2025-10-10

**Authors:** Xinran Liu, Yalin Zhou, Valeria Calcaterra, Gianvincenzo Zuccotti

**Affiliations:** ^1^Department of Clinical Nutrition, Peking University People's Hospital, Beijing, China; ^2^Department of Nutrition and Food Hygiene, School of Public Health, Peking University, Beijing, China; ^3^Pediatric and Adolescent Unit, Department of Internal Medicine, University of Pavia, Pavia, Italy; ^4^Pediatric Department, Buzzi Children's Hospital, Milan, Italy; ^5^Department of Biomedical and Clinical Science, University of Milan, Milan, Italy

**Keywords:** maternal, infant, nutrition, breast milk, gut microbiota, health

## 1 Introduction

Maternal and infant nutrition during pregnancy, lactation, and the early postnatal period exerts a profound influence on child health trajectories. Human milk provides not only macronutrients but also bioactive compounds and microorganisms that shape infant gut microbiota, immune maturation, and metabolic programming. The first 1,000 days of life represent a critical window during which dietary and environmental exposures markedly affect growth, neurodevelopment, and long-term disease risk.

To address these intersections, the Research Topic *Maternal and infant nutrition: impact on breast milk, infant gut microbiota and health development* was launched to explore how maternal diet, milk composition, microbial colonization, and feeding practices converge to influence infant health. Twelve manuscripts were accepted following rigorous peer review, authored by over 60 researchers from Europe, Africa, Asia, and the Americas. These contributions span original research, systematic and narrative reviews, a meta-analysis, and a health policy case study, offering comprehensive insights from mechanistic to translational and public health perspectives. This Research Topic garnered notable engagement, with over 21,000 views and 3,465 downloads within the first months of publication. Such attention underscores both its scientific significance and translational potential. Together, the 12 articles collectively elucidate how maternal nutrition and early feeding strategies influence microbial, neurodevelopmental, and health trajectories in early life.

## 2 Summary of articles

### 2.1 Breast milk composition: source of bioactive components and modulator of infant gut microbiota

Qi et al. analyzed maternal dietary iron intake in relation to neonatal gut microbiota composition in 95 mother–infant dyads. Using 16S rRNA sequencing, they found that higher maternal iron intake during pregnancy was associated with increased microbial diversity, particularly among infants delivered vaginally. Beta-diversity analysis confirmed that iron explained ~10% of the variance in microbial composition. These findings suggest that maternal micronutrient intake during pregnancy exerts measurable programming effects on neonatal gut ecology, potentially influencing immune and metabolic development.

Xiu et al. piloted oropharyngeal therapy with mother's own milk (OPT-MOM) in very preterm infants admitted to neonatal intensive care unit (NICU). Serial oral and fecal samples were collected, and microbial dynamics were tracked over time. Results indicated a reduction in Proteobacteria and enrichment in Firmicutes, with associated beneficial metabolomic changes. These findings suggest that even small volumes of colostrum, when administered oropharyngeally, may positively influence microbial colonization and metabolic development.

Ge et al. investigated the relationship between human milk oligosaccharides (HMOs) and the milk microbiota in colostrum (1–5 days postpartum) and mature milk (42 days postpartum) from 40 mothers. HMO concentrations were significantly higher in colostrum (9.7 g/L) compared to mature milk (6.8 g/L). Specific HMOs, such as 2′-FL, 3′-SL, 6′-SL, and LNT2, decreased over time, while 3′-FL and DFLNH increased. Microbiota diversity and structure also shifted, with Proteobacteria more abundant in colostrum and Firmicutes/Actinobacteria enriched in mature milk. Keystone genera included Serratia, Streptococcus, and Staphylococcus, showing significant correlations with HMOs. Functional predictions indicated reduced infectious disease pathways in mature milk. The study highlights breast milk as a synbiotic system where HMOs and microbes jointly shape infant gut colonization and immunity.

### 2.2 Nutritional interventions and feeding practices: efficacy of dose, timing, and the critical window

Zhang et al. used a controlled rat model to explore the effects of prophylactic vs. therapeutic iron supplementation. Pregnant rats either received supplementation throughout gestation or only after iron-deficiency anemia was established. Preventive supplementation improved iron metabolism and fetal iron markers but failed to normalize maternal hemoglobin. In contrast, post-anemia supplementation significantly improved maternal anemia indices and enhanced fetal length-for-age. This study underscores the importance of timing in micronutrient interventions during pregnancy.

Bendahmane et al. presented a study evaluating the effects of maternal oral supplementation with *Saccharomyces boulardii* CNCM I-1079 during gestation and early lactation on the growth and metabolic profile of newborn puppies. Seventeen bitches and 81 puppies were enrolled, divided into a placebo and a supplemented group. Puppies from supplemented mothers showed a significantly higher early growth rate (12% vs. 7%) during the first 2 days of life. Metabolomic analyses (LC-MS) of urine and serum identified 29 discriminating metabolites, mainly involved in nitrogen metabolism. Notably, urinary proline abundance was positively correlated with growth rate. These findings suggest that maternal supplementation with *S. boulardii* may enhance neonatal growth and survival by modulating nitrogen metabolism pathways.

Yang et al. studied a cohort of preterm infants and assessed brain function using functional near-infrared spectroscopy (fNIRS) and clinical neurological examinations. Infants receiving >70%, and especially >90%, of their enteral intake from mother's own milk showed significantly improved functional connectivity and higher neurological scores. These findings support a strong dose–response effect of breast milk intake on early brain development.

Zhao et al. conducted a large multicenter cohort study to evaluate growth and neurodevelopmental outcomes up to 2 years of corrected age. They found that the benefits of mother's milk intake were most evident in female infants, who showed improved growth trajectories and developmental scores. Male infants showed smaller, though still positive, associations. This highlights possible sex-specific sensitivities to early nutritional exposures.

### 2.3 Shaping supportive environments: policy and systems change for early-life health

Bankole and Li presented a narrative review on the early-life gut microbiome, emphasizing its role in shaping immune, metabolic, and neurological systems during infancy and childhood. The authors describe how the maturation of gut microbial communities is influenced by delivery mode, breastfeeding, diet, antibiotic exposure, physical activity, and environment. Early-life dysbiosis is linked to pediatric diseases, including allergies, gastrointestinal disorders (inflammatory bowel disease, irritable bowel syndrome), obesity, type 1 diabetes, autism spectrum disorders, and Attention-Deficit/ Hyperactivity Disorder (ADHD). The review also highlights therapeutic strategies, such as balanced nutrition, prebiotics, probiotics, synbiotics, postbiotics, microbiota transplantation, and healthy lifestyle interventions, that may restore microbial balance and reduce disease risk, improving long-term health outcomes.

A systematic review by Lu et al. synthesized data from economic evaluations of breastfeeding promotion and human milk-based diets in neonatal intensive care. The majority of studies reported human milk as cost-effective or cost-saving, especially when exclusive human milk diets were implemented. Savings were largely due to reductions in necrotizing enterocolitis, sepsis, and rehospitalization costs. Despite methodological variability, the evidence consistently supported the economic efficiency of investing in human milk feeding in preterm populations.

Brettschneider et al. described the development and implementation of Germany's national breastfeeding strategy. This initiative was structured around seven strategic areas, including professional training, support in healthcare facilities and workplaces, regulation of breast milk substitutes marketing, and systematic monitoring. The participatory process ensured alignment with stakeholders and families, providing a transferable blueprint for other countries.

A mini-review by Swanson et al. offered practical, evidence-based guidance on complementary feeding. It stressed the importance of introducing solid foods gradually, respecting infants' hunger and satiety cues, and fostering a positive social environment around mealtimes. Responsive feeding was emphasized as a critical determinant of healthy growth and eating behaviors. The review also highlighted how early feeding practices influence microbiota development and may mitigate long-term risks of overweight and metabolic disease.

Elema et al. conducted a systematic review and meta-analysis including data from 2000 to 2019. They estimated a pooled prevalence of stunting of 40% among Ethiopian children under five. Determinants included male sex, low birth weight, maternal undernutrition, diarrheal illness, lack of colostrum feeding, and short duration of breastfeeding. The authors highlighted that tackling stunting requires integrated maternal and infant nutrition strategies as well as structural improvements in healthcare and education.

Maccarini et al. comprehensively reviewed the critical role of the exposome—encompassing general external (e.g., air pollution, urbanization), specific external (e.g., nutrition, socioeconomic status), and internal exposures (e.g., metabolic responses)—in shaping the risk of childhood obesity. Notably, the study reinforces the importance of the first 1,000 days as a pivotal window for intervention and calls for interdisciplinary efforts to decode gene-environment interactions. Its findings emphasizing that combating obesity requires a holistic understanding of how environmental cues synergistically influence early metabolic development.

## 3 Emerging perspectives

Collectively, these studies convey that both the quantity and quality of maternal nutrition and milk provision critically shape developmental outcomes. High doses of mother's own milk in preterm infants enhance brain connectivity and developmental functioning, with evidence of sex-specific responsiveness. Maternal micronutrition, including iron intake and probiotic supplementation, can program the neonatal microbiome and metabolic milieu. Delivery mode and early feeding routes, such as OPT-MOM, further influence milk bioactivity and microbial colonization patterns. From a public health standpoint, human milk-based interventions are economically justified in NICUs, and structured policy strategies and caregiver education are essential to support optimal maternal and infant nutrition. Moreover, persistent malnutrition challenges in low-resource settings underscore the need for integrated interventions addressing biological, behavioral, and environmental determinants.

## 4 Conclusions

This Research Topic reaffirms the foundational role of maternal and infant nutrition in influencing growth, neurodevelopment, microbiota, and long-term health. Evidence supports dose-dependent benefits of breastfeeding, the programming potential of maternal nutrition, and the feasibility of early interventions like OPT-MOM. Translational pathways are strengthened by health-economic and policy studies illustrating the feasibility of scaling supportive strategies. Future research should incorporate longitudinal, multi-omic approaches, focus on sex-specific developmental periods, and evaluate economic and societal impacts to ensure evidence-based practices inform both clinical care and public health policy. Protecting and promoting breastfeeding and optimal complementary feeding remains a universal imperative.

